# Defining the DNA Binding Site Recognized by the Fission Yeast Zn_2_Cys_6_ Transcription Factor Pho7 and Its Role in Phosphate Homeostasis

**DOI:** 10.1128/mBio.01218-17

**Published:** 2017-08-15

**Authors:** Beate Schwer, Ana M. Sanchez, Angad Garg, Debashree Chatterjee, Stewart Shuman

**Affiliations:** a Department of Microbiology and Immunology, Weill Cornell Medical College, New York, New York, USA; b Molecular Biology Program, Sloan-Kettering Institute, New York, New York, USA; Harvard Medical School

**Keywords:** DNA binding, fission yeast, phosphate homeostasis, transcriptional regulation

## Abstract

Fission yeast phosphate homeostasis entails transcriptional induction of genes encoding phosphate-mobilizing proteins under conditions of phosphate starvation. Transcription factor Pho7, a member of the Zn_2_Cys_6_ family of fungal transcription regulators, is the central player in the starvation response. The DNA binding sites in the promoters of phosphate-responsive genes have not been defined, nor have any structure-function relationships been established for the Pho7 protein. Here we narrow this knowledge gap by (i) delineating an autonomous DNA-binding domain (DBD) within Pho7 that includes the Zn_2_Cys_6_ module, (ii) deploying recombinant Pho7 DBD in DNase I footprinting and electrophoretic mobility shift assays (EMSAs) to map the Pho7 recognition sites in the promoters of the phosphate-regulated *pho1* and *tgp1* genes to a 12-nucleotide sequence motif [5′-TCG(G/C)(A/T)xxTTxAA], (iii) independently identifying the same motif as a Pho7 recognition element via *in silico* analysis of available genome-wide ChIP-seq data, (iv) affirming that mutations in the two Pho7 recognition sites in the *pho1* promoter efface *pho1* expression *in vivo*, and (v) establishing that the zinc-binding cysteines and a pair of conserved arginines in the DBD are essential for Pho7 activity *in vivo*.

## IMPORTANCE

Fungi respond to phosphate starvation by inducing the transcription of a set of phosphate acquisition genes that comprise a phosphate regulon. Pho7, a member of the Zn_2_Cys_6_ family of fungal transcription regulators, is the central player in the phosphate starvation response in fission yeast. The present study identifies a 12-nucleotide Pho7 DNA binding motif [5′-TCG(G/C)(A/T)xxTTxAA] in the promoters of phosphate-regulated genes, pinpoints DNA and protein features important for Pho7 binding to DNA, and correlates them with Pho7-dependent gene expression *in vivo*. The results highlight distinctive properties of Pho7 vis-a-vis other fungal zinc binuclear cluster transcription factors as well as the divergent cast of transcription factors deployed for phosphate homeostasis in fission yeast versus budding yeast.

## INTRODUCTION

Phosphate homeostasis in the fission yeast *Schizosaccharomyces pombe* is achieved by regulating the transcription of genes encoding three proteins involved in extracellular phosphate mobilization and uptake: a cell surface acid phosphatase, Pho1; an inorganic phosphate transporter, Pho84; and a glycerophosphate transporter, Tgp1 (1). Expression of the genes coding for these proteins is repressed during growth in phosphate-rich medium and induced during phosphate starvation. Induction of the phosphate-regulated genes during starvation depends on transcription factor Pho7 (1–3), a member of the zinc binuclear cluster family of fungal DNA-binding transcription regulators (4).

System-wide microarray analyses of gene expression in *pho7*^+^ and *pho7*Δ strains and chromatin immunoprecipitation sequencing (ChIP-seq) analyses of DNA occupancy by Pho7-TAP in phosphate-replete and phosphate-starved cells affirmed the role of Pho7 in the phosphate starvation response, while highlighting an additional function in driving expression of multiple stress response genes independent of phosphate status (1).

We have shown that a 283-nucleotide (nt) segment of DNA 5′ of the *pho1* transcription start site suffices to drive expression of Pho1 acid phosphatase activity *in vivo*. Because the 283-nt *pho1* promoter generated no acid phosphatase activity in a *pho7*Δ strain, this DNA segment must include the requisite Pho7 binding site(s) (5). Genome-wide ChIP-seq analysis revealed a peak of Pho7-TAP occupancy from positions −230 to −120 upstream of the *pho1* transcription start site (1). However, the DNA element recognized by Pho7 remains unknown, and prior attempts to identify a DNA binding motif based on genome-wide Pho7 ChIP-seq data were unsuccessful (1).

In the present study, we define the DNA site recognized by Pho7, via two independent approaches: (i) a revisited *in silico* analysis of the Pho7 ChIP-seq data and (ii) biochemical footprinting of the Pho7 DNA-binding domain (DBD) on the *pho1* and *tgp1* gene promoters. We identify DNA features important for Pho7 binding and correlate them with Pho7-dependent gene expression. Our results provide new insights into the fission yeast phosphate response and illuminate distinctive properties of Pho7 vis-a-vis other fungal zinc binuclear cluster transcription factors.

## RESULTS

### The zinc-binding cysteines are essential for Pho7 activity *in vivo*.

**FIG 1:**
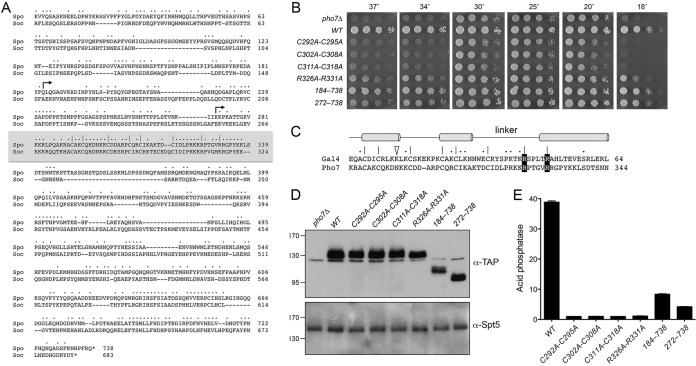
Features of Pho7 essential for its activity *in vivo*. (A) The amino acid sequence of *S. pombe* Pho7 (Spo) is aligned to the sequence of the homologous protein from *S. octosporus* (Soc). Gaps in the alignment are denoted by dashes. Positions of side-chain identity/similarity are denoted by dots. The putative Zn_2_Cys_6_ DNA-binding domain is highlighted in the gray box. Conserved pairs of cysteines and arginines in the DBD that were targeted for alanine substitution are indicated by |. The margins of the N-terminal deletions are indicated by arrowheads. (B) *S. pombe* strains deleted of *pho7* (*pho7*Δ) or bearing the *pho7* alleles as indicated were spot tested for growth at the temperatures specified. (C) Similarity of the Zn_2_Cys_6_ DNA-binding domains of *S. cerevisiae* Gal4 and *S. pombe* Pho7. The secondary structure elements of Gal4 are indicated above the amino acid sequence, with α-helices depicted as horizontal cylinders. The signature zinc-binding cysteines are indicated by |. Conserved arginines that in Gal4 contact the DNA phosphodiester backbone are shown in white font on a black background. Other positions of side-chain identity/similarity are denoted by dots. The Gal4 lysine that contacts adjacent guanine nucleobases in the DNA target site is indicated by a triangle. (D) Western blots of whole-cell extracts prepared from *S. pombe* strains with the indicated *pho7* alleles. The blots were probed with antibodies recognizing the TAP tag or the Spt5 protein. (E) Acid phosphatase activity of cells bearing the indicated *pho7-TAP* alleles assayed 5 h after transfer of logarithmically growing cells to medium lacking phosphate.

The 738-amino-acid (aa) *S. pombe* Pho7 protein is most closely related to its 683-aa homolog from *Schizosaccharomyces octosporus*. Alignment of the two polypeptides highlights 291 positions of side-chain identity/similarity across their full lengths, with the highest conservation being in the central Zn_2_Cys_6_ domain (aa 282 to 339 in Pho7 [shaded gray in Fig. 1A]) and the C-terminal segment (aa 629 to 722 in Pho7). To gauge the role of the putative zinc-binding cysteines in Pho7 function, we replaced the chromosomal *pho7*^+^ gene with a *pho7*-*TAP*::*hygMX* cassette (encoding a C-terminal TAP-tagged Pho7 polypeptide) in which *pho7* was either wild type (WT) or had double-alanine mutations of three serial cysteine pairs (*C292A-C295A*, *C302A-C308A*, and *C311A-C318A*). Spot testing for growth on YES (yeast extract with supplements) agar medium showed that the *pho7*Δ strain grew slower than wild type at 20, 25, and 30°C (as gauged by colony size) and was extremely sick at higher (34 and 37°C) and lower (18°C) temperatures (Fig. 1B). All three of the double-cysteine mutations phenocopied the growth defect of the *pho7*Δ strain, suggesting that zinc binding by Pho7 is essential for Pho7 function in sustaining growth at extremes of temperature.

The central Zn_2_Cys_6_ domain of Pho7 is homologous to the N-terminal Zn_2_Cys_6_ DNA-binding domain (DBD) of *Saccharomyces cerevisiae* Gal4, a prototypal fungal zinc cluster transcription factor. The DBD consists of a folded zinc finger module of two α-helices that recognizes the target DNA sequence, a linker that contacts the DNA phosphate backbone, and a helical coiled-coil module that mediates dimerization of the DBD (6, 7) (Fig. 1C). It is noteworthy that the Lys18 side chain in Gal4 that makes hydrogen bonds to the adjacent guanine nucleobases in the major groove of the CGG target sequence (indicated by the open triangle in Fig. 1C) is not conserved in Pho7 (where the corresponding residue is Asn299). However, the Gal4 Arg46 and Arg51 side chains (located in the linker and at the beginning of the dimerization α-helix, respectively) that contact target site DNA phosphates are conserved in Pho7 as Arg326 and Arg331 (highlighted in white font on a black background in Fig. 1C). We found that a strain with an *R326A-R331A* double-alanine mutation in *pho7* grew better than the *pho7*Δ null strain at high and low temperatures, although not as well as the wild type (Fig. 1B), indicating that the loss of putative interactions of these conserved arginines with DNA elicits a hypomorphic phenotype during vegetative growth.

Protein segments flanking the DBD of fungal zinc cluster proteins are involved in transcription activation and responses to regulatory inputs. Guided by the alignment of Pho7 with its *S. octosporus* homolog, we deleted either the N-terminal 183 or 271 aa and replaced *pho7*^+^ with the *pho7*(*184–738*)-*TAP* and *pho7*(*272–738*)*-TAP* alleles. Both truncation strains grew like the wild type at 18 to 30°C; the truncation strains grew better than the *pho7*Δ mutant at 34 and 37°C, albeit with colony sizes slightly smaller than wild type (Fig. 1B).

Whole-cell extracts of the *pho7-TAP* strains were subjected to SDS-PAGE and Western blotting with anti-TAP antibody (Fig. 1D). Western blotting with antibody to *S. pombe* Spt5 was used as a loading control. An immunoreactive ~130-kDa polypeptide corresponding to Pho7-TAP was detected in extracts from the *pho7-TAP* strains but not in extracts from *pho7*Δ cells. TAP-reactive Pho7 polypeptides of serially smaller molecular masses were detected in extracts of the two truncation strains (Fig. 1D).

The *pho7-TAP* strains were tested for responsiveness to phosphate starvation, by assay of cell surface Pho1 acid phosphatase activity 5 h after transfer of the cells from rich medium to synthetic medium lacking phosphate. The *C292A-C295A*, C*302A-C308A*, *C311A-C318A*, and *R326A-R331A* strains manifested no Pho1 induction during phosphate starvation (Fig. 1E). Thus, disruption of the zinc-binding cluster or loss of the pair of putative DNA-binding arginines eliminates Pho7 function in phosphate homeostasis. The extents of Pho1 induction by the *pho7*(*184–738*) and *pho7*(*272–738*) truncation strains were 22% and 11% of that of the wild type, respectively (Fig. 1E).

### *In silico* identification of a candidate Pho7 recognition element.

**FIG 2:**
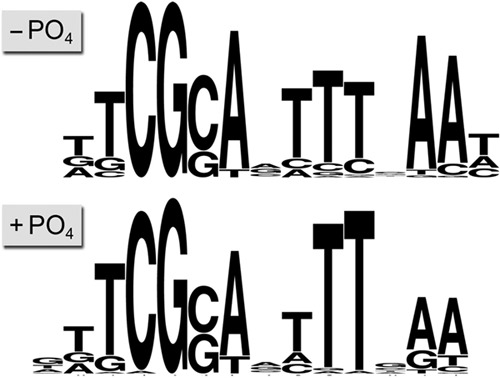
*In silico* identification of a candidate Pho7 recognition element. Motifs were identified via analysis of previously reported Pho7-TAP ChIP-seq data (1) from phosphate-replete and phosphate-starved cells as described in the text.

The available genome-wide Pho7-TAP ChIP-Seq data from phosphate-replete and phosphate-starved cells (1) (GEO accession no. GSE39498) were processed for Pho7-TAP peak localization above background, and the peak sequences were screened for enriched motifs in MEME (8) as described in Materials and Methods. The separate analyses of the phosphate-starved and phosphate-replete data sets yielded virtually identical candidate motifs, with E values of 1.6 × 10^−15^ and 9.6 × 10^−14^, respectively (Fig. 2). The consensus motif for the two data sets is a dodecamer, 5′-TCG(G/C)AxTTTxAA. Occurrences of this motif within individual Pho7-TAP ChIP peaks as determined in FIMO (9) and the identities of *S. pombe* genes within 1 kb of the peaks are compiled in Tables S1 and S2, respectively, in the supplemental material.

10.1128/mBio.01218-17.1 TABLE S1  Individual motif occurrences within the sequences of the Pho7 ChIP peaks, identified by FIMO (9), are listed (cutoff, motif *P* value of ≤0.001) on separate pages for phosphate-starved (−P_i_) and phosphate-replete (+P_i_) conditions. The motifs that correlate with the Pho7 DBD DNase I footprints in the *pho1* and *tgp1* promoters are highlighted in yellow. Download TABLE S1, XLSX file, 0.1 MB. Copyright © 2017 Schwer et al. 2017 Schwer et al. This content is distributed under the terms of the Creative Commons Attribution 4.0 International license.

10.1128/mBio.01218-17.2 TABLE S2  Annotated genes located within 1 kb downstream of the Pho7 ChIP peaks (group 1) and those that have a Pho7 ChIP peak within the annotated gene (group 2) are listed on separate pages for phosphate-starved (−P_i_) and phosphate-replete (+P_i_) conditions. Download TABLE S2, XLSX file, 0.1 MB. Copyright © 2017 Schwer et al. 2017 Schwer et al. This content is distributed under the terms of the Creative Commons Attribution 4.0 International license.

### Recombinant Pho7 Zn_2_Cys_6_ DBD binds and footprints the *pho1* promoter.

**FIG 3:**
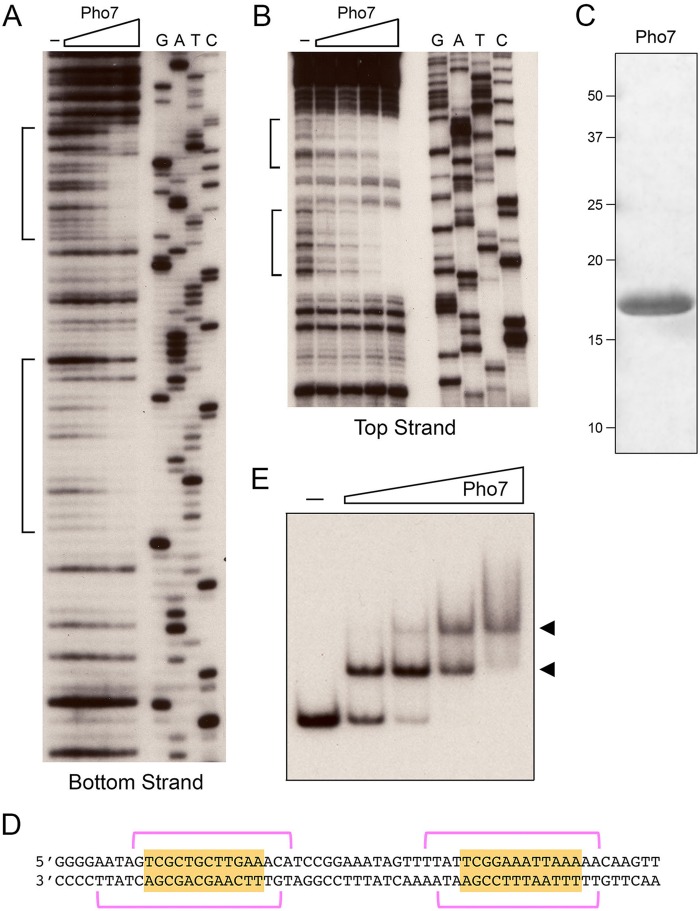
Recombinant Pho7 Zn_2_Cys_6_ DBD binds and footprints the *pho1* promoter. (A and B) DNase I footprint analyses of the bottom strand (A) and top strand (B) of the *pho1* promoter are shown. Binding reaction mixtures contained 0.75 pmol of 5′ ^32^P-labeled DNA and either no added Pho7 DBD (lanes –) or increasing amounts of Pho7 DBD (13, 17, 22, 33, and 66 ng in panel A or 20, 27, 40, and 80 ng in panel B). The DNase I digestion products were analyzed by denaturing PAGE in parallel with a series of DNA-directed primer extension reactions (using a ^32^P-labeled primer identical to the labeled 5′ end of the footprinted DNA) that contained mixtures of standard and chain-terminating nucleotides. (The chain terminator is specified above the lanes.) The margins of the footprints are indicated by brackets. (C) SDS-PAGE analysis of recombinant Pho7 DBD. The Coomassie blue-stained gel is shown. The positions and sizes (in kilodaltons) of marker polypeptides are indicated on the left. (D) Nucleotide sequence of the top and bottom strands of the *pho1* promoter from −196 to −136 upstream of the transcription start site. The DNase I footprints on each strand are denoted by magenta brackets. A conserved dodecamer sequence within the footprints is highlighted in gold. (E) Gel shift assay on Pho7 binding to the *pho1* promoter. Reaction mixtures (10 µl) containing 1 pmol of a 160-nt ^32^P-labeled DNA fragment, 0.34 µg poly(dI-dC), and either no protein (–) or increasing amounts of Pho7 DBD (8.3, 17, 33, or 66 ng) were incubated for 10 min at room temperature and then analyzed by native PAGE. An autoradiograph of the gel is shown. The arrowheads on the right indicate positions of DNA-protein complexes.

Pho7(279–368), which spans the conserved Zn_2_Cys_6_ domain, was produced in *Escherichia coli* as a His_10_-Smt3 fusion protein and isolated from a soluble bacterial extract by nickel affinity chromatography. The His_10_-Smt3 tag was removed with the Smt3-specific protease Ulp1, and the native Pho7(279–368) protein (henceforth referred to as Pho7 DBD) was separated from the tag by a second round of nickel affinity chromatography and purified further by gel filtration. SDS-PAGE analysis revealed a predominant polypeptide that migrated at ~17 kDa relative to size standards (Fig. 3C), although the calculated size of the recombinant polypeptide is 10 kDa. An initial DNase I footprinting experiment was performed using a 300-bp 5′ ^32^P-labeled DNA fragment comprising 249 nt upstream of the *pho1* transcription start site and extending 51 nt into the transcription unit. The DNA was end labeled on the top DNA strand. Limited DNase I digestion in the absence and presence of increasing levels of Pho7 DBD revealed two segments of protection (denoted by brackets in Fig. 3B) separated by an unprotected spacer segment. Analysis in parallel with a 5′ ^32^P-labeled primer extension sequencing ladder (Fig. 3B) demarcated the two regions of DNase I protection as −188 to −173 and −159 to −142 upstream of the *pho1* transcription start site (Fig. 3D). We proceeded to footprint the bottom DNA strand using a 160-bp DNA fragment from nt −249 to −90 upstream of the *pho1* transcription start site. Here we again observed two segments of DNase I protection (Fig. 3A, denoted by brackets). Reference to a 5′ ^32^P-labeled primer extension sequencing ladder (Fig. 3A) assigned the two bottom strand DNase I footprints as −192 to −174 and −158 to −142 upstream of the *pho1* transcription start site, respectively (Fig. 3D). Both of the Pho7 footprint sites include a 12-nt motif, 5′-TCG(G/C)(A/T)xxTTxAA (Fig. 3D), that resembles the candidate Pho7 recognition element identified via our *in silico* analysis (Fig. 2).

We also assessed binding of Pho7 DBD to the *pho1* promoter by electrophoretic mobility shift assay (EMSA) using the 160-bp 5′ ^32^P-labeled DNA probe comprising nt −249 to −90 upstream of the *pho1* transcription start site. Pho7 DBD formed a single discrete DNA-protein complex at lower Pho7 DBD concentrations that was converted to a more slowly migrating complex as the Pho7 DBD concentration was increased (Fig. 3E). The evolution of the DNA-protein complexes as a function of protein concentration is indicative of the sequential (noncooperative) mode of Pho7 binding to the two sites footprinted in the *pho1* promoter. If binding had been highly cooperative, we would have expected to see accumulation of the two-site-occupancy DNA-protein complexes at protein concentrations at which there was a significant fraction of residual unbound DNA.

**FIG 4:**
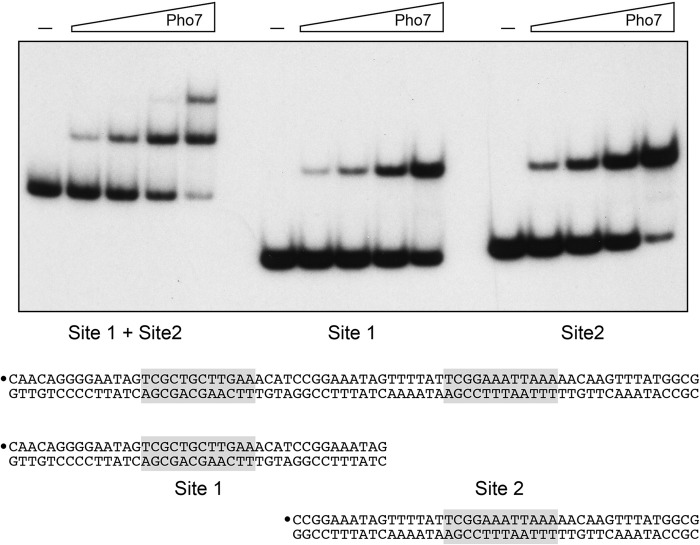
Pho7 DBD binds independently to two target sites in the *pho1* promoter. The sequences of three *pho1* promoter DNAs containing both Pho7 binding sites or individual Pho7 binding sites are shown at the bottom. The consensus motifs are shaded in gray, and the 5′ ^32^P labels on the top strands are indicated by large dots. Reaction mixtures (10 µl) containing 1 pmol ^32^P-labeled DNA, 0.34 µg poly(dI-dC), and either no protein (–) or increasing amounts of Pho7 DBD (8.3, 17, 33, or 66 ng) were incubated for 10 min at room temperature. The mixtures were analyzed by native PAGE. An autoradiograph of the gel is shown.

To query whether the Pho7 DBD requires tandem recognition sites or binds independently to each of the target sites in the *pho1* promoter, we performed EMSAs with a 5′ ^32^P-labeled 73-bp DNA probe spanning both sites and with 40- and 43-bp DNA probes corresponding to only site 1 and site 2, respectively (Fig. 4). The results showed that Pho7 binds sequentially to two sites in the 73-bp DNA to give rise to two discrete shifted complexes and binds independently to site 1 and site 2 DNAs to form a single shifted complex in each case (Fig. 4). Quantification of the yield of the Pho7-DNA complex as a function of input protein indicated that Pho7 had 2-fold-higher affinity for site 2 than for site 1.

### Effect of Pho7 site mutations in the *pho1* promoter on Pho1 expression *in vivo*.

**FIG 5:**
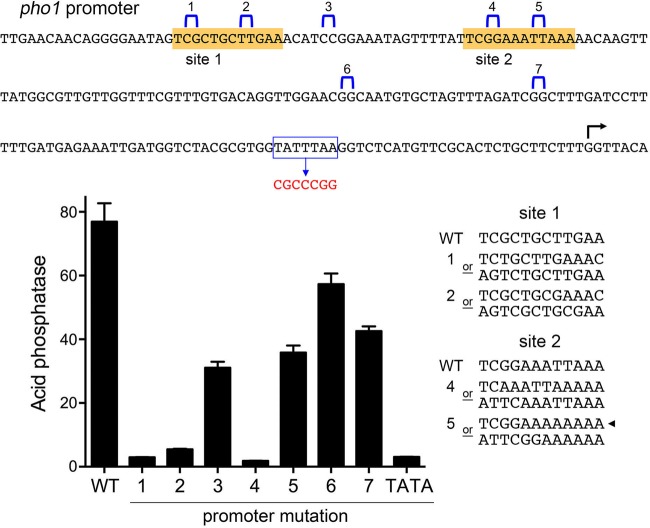
Effect of Pho7 site mutations in the *pho1* promoter on Pho1 expression *in vivo*. The sequence of the *pho1* promoter from −206 to +7 relative to the *pho1* transcription start site (denoted by an arrowhead) is shown at the top. Pho7 binding sites 1 and 2 are shaded in gold, and dinucleotides that were deleted in promoter mutations 1 to 7 are marked by blue brackets above the sequence. A putative TATA element (outlined in blue) was replaced by the sequence shown in red. The sequences at right in the bottom panel show the two possibilities of how the dinucleotide deletions alter the Pho7 binding motifs for site 1 and site 2. The arrowhead indicates the more likely scenario of how promoter mutation 5 alters the Pho7 binding site. Reporter plasmids with wild-type and the mutated *pho1* promoters were introduced into a strain with the endogenous *pho1* gene deleted. Plasmid-containing cells were grown logarithmically in YES medium and assayed for acid phosphatase activity.

A plasmid-based reporter containing 283 nt of promoter DNA 5′ of the *pho1* transcription start site suffices to drive expression of Pho1 acid phosphatase in fission yeast cells in which the chromosomal *pho1*^+^ locus has been deleted (5). To gauge the role of the Pho7 binding sites in *pho1* promoter activity, we made a series of promoter mutants in this plasmid in which dinucleotides were deleted, either within the margins of the Pho7 footprints (mutants 1, 2, 4, and 5), between the two Pho7 sites (mutant 3), or between the Pho7 sites and a putative TATA box element, ^−34^TATTTAA^−28^, preceding the transcription start site (mutants 6 and 7). The deleted dinucleotides are indicated by brackets above the *pho1* promoter sequence in Fig. 5. In parallel, we replaced the TATTTAA sequence with CGCCCGG. *pho1*Δ cells carrying the wild-type and promoter mutant plasmids were assayed for acid phosphatase activity. Mutation of the TATA box sequence effaced Pho1 expression, as did the dinucleotide deletions 1, 2, and 4 within the Pho7 binding sites (Fig. 5). Each of these dinucleotide deletions alters the Pho7 site by shifting the flanking DNA sequences to create two possible mutant versions of the dodecamer (indicated by “or” in Fig. 5), both of which deviate greatly from the Pho7 site consensus. However, deletion 5 within Pho7 site 2 had only a modest effect, reducing Pho1 expression to 47% of the wild-type value. We attribute the residual activity to the fact that one of the frameshifted versions of site 2 mutant 5 (indicated by the arrowhead in Fig. 5) retains many of the consensus nucleotides of the Pho7 dodecamer 5′-TCG(G/C)AxTTTxAA while replacing the triplet of T’s with A’s. The dinucleotide promoter deletions 3, 6, and 7 supported 40, 75, and 55% of wild-type Pho1 expression, respectively. These experiments highlight that both Pho7 sites and the TATA box are critical for *pho1* promoter activity *in vivo*.

### Pho7 binding site in the *tgp1* promoter.

**FIG 6:**
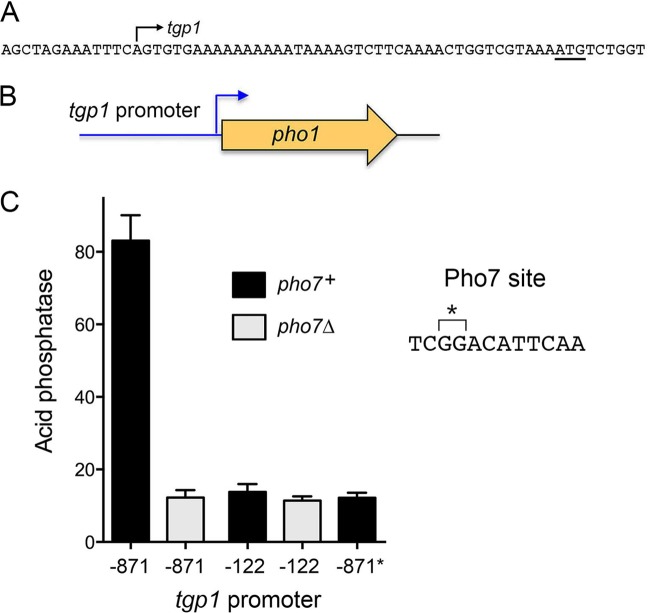
Plasmid reporter of *tgp1* promoter function. The DNA sequence from positions −13 to +51 relative to the *tgp1* transcription start site (indicated by the arrow) is shown. The *tgp1* ATG translation start site is underlined. (B) In the *tgp1-pho1* reporter plasmids, the *pho1* ORF was fused immediately downstream of a fragment of genomic DNA encompassing the *tgp1* transcription start site plus 42 bp of downstream sequences and 871 bp (−871) or 122 bp (−122) of upstream *tgp1* sequences. (C) Acid phosphatase activity of *S. pombe pho1*Δ strains bearing the indicated *tgp1-pho1* reporter plasmids during logarithmic growth in YES medium. The strains were either *pho7*^+^ or *pho7*Δ as specified. A dinucleotide deletion (*) in the Pho7 binding site, indicated by a bracket in the sequence at right, was introduced in the context of the −871 *tgp1-pho1* reporter (−871*).

We employed reverse transcriptase primer extension analysis to locate the 5′ end of the *tgp1* mRNA. A ^32^P-labeled DNA primer complementary to nt 2 to 23 of the of the *tgp1* open reading frame (ORF) was annealed to RNA isolated from phosphate-starved yeast cells and then subjected to reverse transcription (RT). The RT primer extension product was analyzed in parallel with a chain-terminated sequencing ladder generated by DNA polymerase extension of the same ^32^P-labeled primer annealed to a DNA template containing the *tgp1* locus (not shown). A single 5′ end was thereby located 42 nt upstream of the start codon of the *tgp1* open reading frame (ORF) (Fig. 6A).

To interrogate the *tgp1* promoter, we constructed a plasmid reporter in which a genomic DNA segment containing nt −871 to +42 of the *tgp1* transcription unit (with +1 being the mRNA start site) was fused to the *pho1* ORF (Fig. 6B). Because this plasmid generated vigorous acid phosphatase activity when introduced into a *pho1*Δ strain (Fig. 6C), we surmised that the 871-nt segment upstream of the transcription start site embraces a *tgp1* promoter and potential regulatory elements. Acid phosphatase activity driven by the *tgp1* promoter reporter plasmid was reduced by 85% in a *pho7*Δ strain background (Fig. 6C), signifying that the 871-nt segment contains a site or sites that are bound by the Pho7 transcription factor.

**FIG 7:**
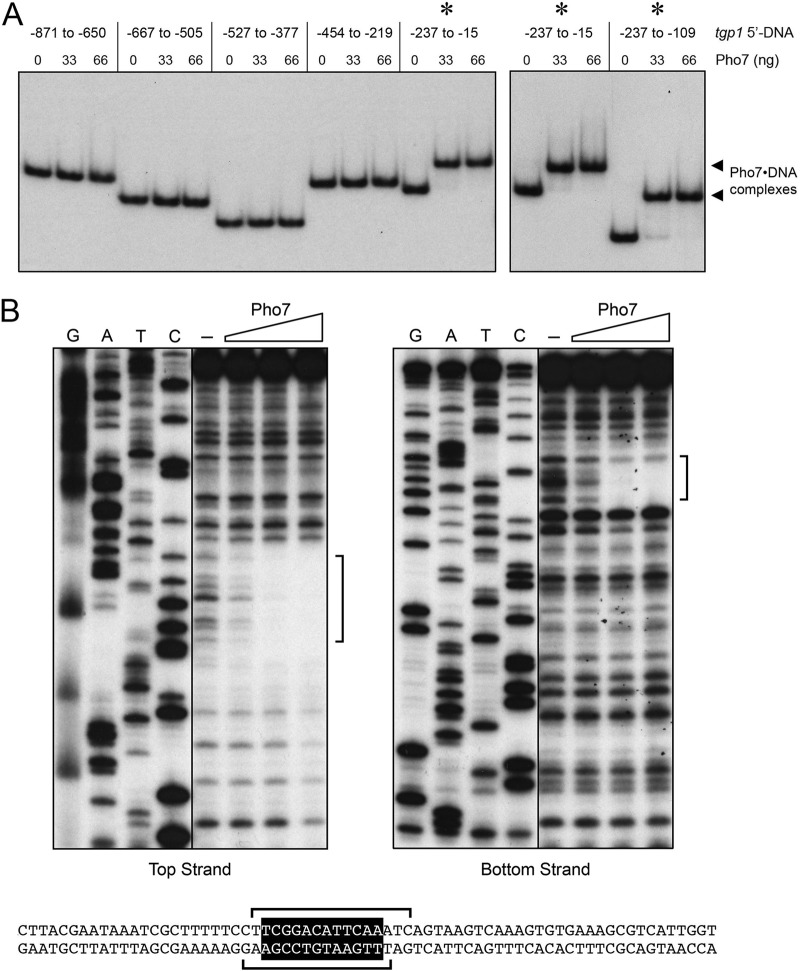
Pho7 binding site in the *tgp1* promoter. (A) EMSAs using ^32^P-labeled DNA fragments embracing the indicated sequences of the *tgp1* locus upstream of the *tgp1* transcription start site (defined as +1). The nucleotide margins of the DNA segments are indicated at the top. Reaction mixtures (10 µl) containing 0.6 pmol labeled DNA, 340 ng poly(dI-dC), and 0, 33, or 66 ng of Pho7 DBD were incubated for 10 min at room temperature. The mixtures were analyzed by native PAGE. An autoradiograph of the gel is shown. The asterisks above the gel denote *tgp1* promoter segments that are bound by Pho7; the arrowheads at right mark the positions of the DNA-protein complexes. (B) DNase I footprinting analyses of the top strand (left panel) and bottom strand (right panel) of the *tgp1* promoter are shown along with the respective sequencing ladders. Binding reaction mixtures contained 1 pmol of 5′ ^32^P-labeled DNA and either no added Pho7 DBD (lanes –) or increasing amounts of Pho7 DBD (12.5, 25, or 50 ng). The margins of the footprints are indicated by brackets. The *tgp1* sequence from −216 to −148 upstream of the *tgp1* transcription start site is shown at the bottom. The Pho7 binding motif is shown in white font on a black background. The brackets denote the DNase I footprints on the top and bottom strands.

To locate such sites, a series of overlapping ^32^P-labeled DNA fragments spanning the genomic region from nt −871 to −15 preceding the transcription start site of the *tgp1* gene was prepared and tested by EMSA for binding to Pho7 DBD. Whereas there was no protein-DNA complex formed in DNA fragments in the region from −871 to −219, the 223-nt DNA fragment from −237 to −15 was bound by Pho7 DBD to form a single protein-DNA complex (Fig. 7A). Pho7 DBD also formed a single protein-DNA complex on a 129-nt DNA fragment from −237 to −109 (Fig. 7A). This DNA segment was used in DNase I footprinting experiments shown in Fig. 7B. A single region of protection from DNase I cleavage spanned nt −193 to −178 on the top strand and nt −194 to −180 on the bottom strand. The footprint is denoted by brackets in Fig. 7B, and it embraces a 12-mer sequence (5′-TCGGACATTCAA) that is identical at 10 of 12 positions to Pho7 recognition site 2 in the *pho1* promoter.

With this information in hand, we truncated the −871 *tgp1* promoter reporter plasmid to position −122, thereby deleting the Pho7 site. Pho1 acid phosphatase activity expressed by the truncated promoter was reduced to 17% of the level driven by the −871 promoter (Fig. 6C), thereby phenocopying the effect of *pho7*Δ on the longer promoter. Indeed, the residual acid phosphatase expression driven by the −122 *tgp1* promoter was maintained in a *pho7*Δ background (Fig. 6C). To query whether the effect of promoter truncation is caused by loss of the Pho7 site, we deleted the GG dinucleotide from the Pho7 site in the context of the −871 *tgp1* promoter; this dinucleotide deletion (−871*) reduced acid phosphatase expression by 85%, mimicking the effects of *pho7*Δ and the promoter truncation (Fig. 6C). We conclude that the Pho7 binding site identified by EMSA and footprinting is essential for Pho7-dependent *tgp1* promoter activity.

### Effect of nucleotide substitutions on Pho7 binding to its DNA target.

**FIG 8:**
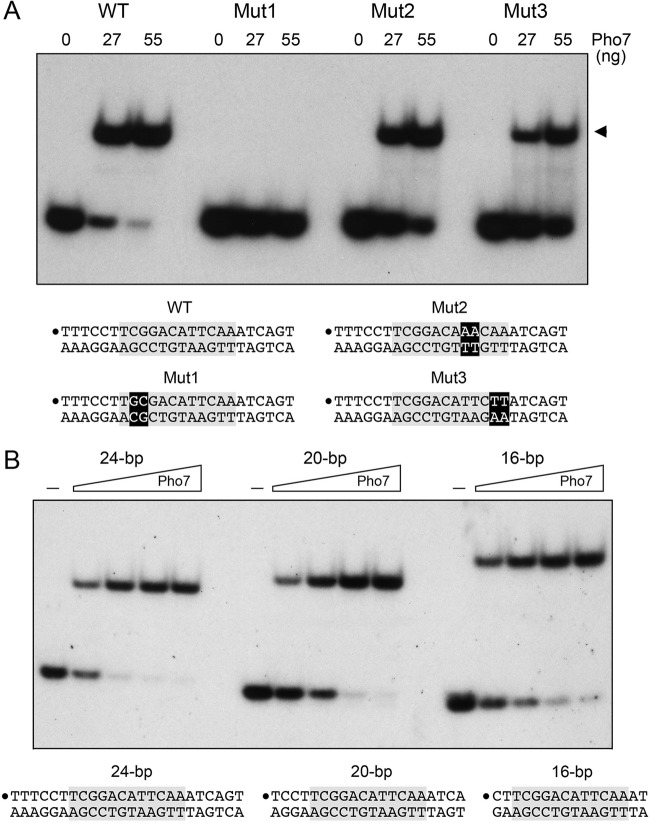
Effect of nucleotide substitutions and deletion of flanking nucleotides on Pho7 binding to its DNA target. EMSAs were performed using the indicated DNA duplexes encompassing the Pho7 binding site in the *tgp1* promoter. Reaction mixtures (10 µl) containing ^32^P-labeled DNAs (1 pmol), 340 ng poly(dI-dC), and Pho7 DBD as specified were incubated for 10 min at room temperature and then analyzed by native PAGE. Autoradiographs of the gels are shown. The sequences of the DNAs are indicated below the gels; the Pho7 binding motif is shaded gray, and the 5′ ^32^P labels on the top strand are indicated by large dots. (A) Effect of nucleotide changes. The two-nucleotide substitutions in Mut1, Mut2, and Mut3 are denoted in white font on a black background. Reaction mixtures contained 0, 27, or 55 ng Pho7 DBD as indicated. (B) Effect of deleting flanking nucleotides. Reaction mixtures contained either no added Pho7 DBD (lanes –) or increasing amounts of Pho7 DBD (8.3, 17, 33, or 66 ng from left to right in the titration series).

The three Pho7 binding sites mapped biochemically in the *pho1* and *tgp1* promoters agree with the consensus 5′-TCG(G/C)AxxTTxAA site identified *in silico*. To understand the role of nucleobase sequence in Pho7 recognition, we prepared a series of 5′ ^32^P-labeled 24-bp DNA duplexes based on the footprinted Pho7 site in the *tgp1* promoter that corresponded to the native wild-type dodecamer DNA sequence 5′-TCGGACATTCAA or had 2-base substitutions (underlined) as follows: 5′-TGCGACATTCAA (Mut1), 5′-TCGGACAAACAA (Mut2), or 5′-TCGGACATTCTT (Mut3). The 24-mers were tested by EMSA for binding to Pho7 DBD (Fig. 8A). DNA binding was effaced by Mut1 that changed the CG dinucleotide at positions 2 and 3 of the dodecamer element to GC. The loss of DNA binding *in vitro* by altering the CG dinucleotide is consistent with the loss of *pho1* expression *in vivo* when the CG (site 1) or the GG (site 2) dinucleotide is altered in the site 1 or site 2 dodecamers of the *pho1* promoter (Fig. 5). In contrast, the Mut2 and Mut3 dinucleotide changes reduced but did not eliminate Pho7 DBD affinity for the *tgp1* promoter site: i.e., 27 ng of input Pho7 DBD sufficed to bind 81% of the wild-type 24-mer DNA but only 45% and 23% of the Mut2 and Mut3 DNAs, respectively (Fig. 8A). It is instructive that the ~2-fold decrease in Pho7 binding *in vitro* elicited by the TT-to-AA change in the dodecamer correlates with the 2-fold decrease in *pho1* expression when the same TT-to-AA change is created in site 2 of the *pho1* promoter (Fig. 5).

To gauge whether DNA flanking the dodecamer plays a role in Pho7 binding, we tested in parallel the 24-bp DNA duplex from the *tgp1* promoter and two shorter versions, a 20-bp duplex and a 16-bp duplex, in which 2 or 4 nt were deleted on both sides of the central dodecamer. The Pho7 DBD bound with similar affinity to all three DNAs (Fig. 8B).

## DISCUSSION

The 738-aa Pho7 protein is the key transcriptional activator underlying fission yeast phosphate homeostasis. Here, we purified a recombinant Pho7 DNA-binding domain (DBD) that includes the Zn_2_Cys_6_ module and used it in DNase I footprinting experiments to map the Pho7 recognition sites in the promoters of the *pho1* and *tgp1* genes to a specific 12-nt motif, 5′-TCG(G/C)(A/T)xxTTxAA. We independently identified the same motif as a Pho7 recognition element via *in silico* analysis of available genome-wide ChIP-seq data (1). The *pho1* promoter contains two Pho7 recognition sites, in direct repeat orientation and separated by a 20-nt spacer. EMSAs showed that the Pho7 DBD binds independently, and apparently noncooperatively, to these two sites in the *pho1* promoter. Two-nucleotide deletions within each of the Pho7 recognition sites sharply reduced Pho1 expression *in vivo*, signifying that both Pho7 sites are needed to activate *pho1* transcription. (The spacing between Pho7 sites 1 and 2 might also be relevant for *pho1* promoter activity, insofar as a dinucleotide deletion between the sites reduced Pho1 expression by half. A systematic interrogation of the effects of contracting and expanding the spacer length is a subject for future studies.) In contrast, the *tgp1* promoter appears to contain only a single Pho7 binding site that is important for Pho7-dependent *tgp1* promoter activity.

The Pho7 DBD resembles the DBDs of other zinc cluster fungal transcription factors with respect to the presence and spacing of its six conserved cysteines. We find that the three pairs of zinc-coordinating cysteines are essential for Pho7 function *in vivo*, as gauged by two criteria: complementation of the conditional growth defects of a *pho7*Δ strain and the ability to mount a Pho1 inductive response to phosphate starvation. We surmise that DNA binding by Pho7 is necessary for its biological activity. The Pho7 DBD also has a pair of arginines that are conserved in Gal4, where they contact the phosphate backbone of the Gal4 DNA recognition site. Mutation of this arginine pair in Pho7 eliminated Pho1 expression during phosphate starvation, again consistent with DNA binding being crucial for Pho7 activity during the starvation response.

In many of the zinc cluster fungal transcription factors, the DBD is located at the very N terminus of the polypeptide (4). This is not the case in Pho7, where the DBD is preceded by a 270-aa segment that is weakly conserved in the Pho7-like protein of *S. octosporus* (Fig. 1A). A truncated Pho7 lacking this N domain complemented the cold-sensitive growth defect of the *pho7*Δ mutant, but only partially complemented the temperature-sensitive (*ts*) growth phenotype. With respect to phosphate homeostasis, deletion of the N domain sharply reduced Pho1 production in response to phosphate starvation. It is noteworthy that eight sites of Pho7 phosphorylation have been annotated within the N domain: S230, S244, S247, S249, S253, S257, T260, and T261 (http://www.pombase.org). Five of these phosphorylation sites are conserved in the *S. octosporus* N domain.

The Pho7 DNA recognition site 5′-TCG(G/C)(A/T)xxTTxAA differs from those of other well-studied fungal Zn_2_Cys_6_ proteins (e.g., Gal4, Leu3, Hap1, Put3, and Ppr1), which typically recognize pairs of CGG triplets that are arranged as inverted, direct, or everted repeats (4, 6, 10–13). The fungal Zn_2_Cys_6_ proteins that bind to such DNA elements typically do so as homodimers in which the zinc-binding module confers DNA sequence recognition and a downstream α-helix forms a coiled-coil dimer interface. The *pho1* promoter site 2 and *tgp1* promoter site that are recognized by Pho7 contain a single CGG triplet, mutation of which to GCG effaces target recognition. The lack of internal symmetry in the Pho7 DNA binding site raises the prospects that (i) it might have a unique binding mode in which the component promoters of a homodimer recognize different nucleotide sequence motifs or (ii) it might bind to DNA as a monomer, *a la* the *Aspergillus* transcription factor AlcR (14). Further insights into Pho7 specificity will hinge on cocrystallizing the DBD bound to target DNA and educing structure-activity relations via comprehensive mutagenesis of the DBD.

Finally, it is worth highlighting that whereas both fission and budding yeasts respond to phosphate starvation by inducing the transcription of phosphate acquisition genes (a phosphate regulon), they rely on quite different classes of DNA-binding transcription factors to achieve this response (2). As affirmed here, the Pho7 protein that drives phosphate homeostasis in *S. pombe* is a member of the fungal Zn_2_Cys_6_ transcription factor family. In contrast, transcription of the phosphate regulon in phosphate-starved *S. cerevisiae* depends on cooperative action of two distinct transcription factors: Pho4 and Pho2 (15–17). Pho2 is a member of the homeodomain family. Pho4 belongs to the basic helix-loop-helix (bHLH) family of transcription factors, and its activity is regulated negatively/positively by phosphorylation/dephosphorylation under phosphate-replete/phosphate-starved conditions (18). The crystal structure of the Pho4 bHLH domain homodimer bound to its 17-bp high-affinity DNA target site revealed a network of amino acid contacts with the nucleobases of the central 7-bp 5′-CACGTGG element (19). There is no similarity in the DNA sequences recognized by *S. pombe* Pho7 and *S. cerevisiae* Pho4. Pho4-like bHLH transcription factors drive the phosphate starvation response in the fungal pathogens *Candida glabrata* and *Cryptococus neoformans* (20, 21). The evolutionary paths that led to the extreme divergence in the choice of transcription factors that govern phosphate homeostasis in fission versus budding yeast are presently obscure.

## MATERIALS AND METHODS

### Allelic exchange at the *pho7* locus.

We constructed a series of pKS-based plasmids carrying *pho7-TAP* integration cassettes marked with *hygMX* by PCR amplification using genomic DNA as a template and oligonucleotides that introduced restriction sites for cloning. The cassettes consisted of the following elements, proceeding from 5′ to 3′: (i) a 515-bp segment of genomic DNA 5′ of the *pho7*^*+*^ start codon; (ii) an open reading frame encoding wild-type Pho7, or mutated versions thereof, fused to a 560-bp ORF encoding the TAP tag (22); (iii) a 302-bp segment of genomic DNA 3′ of the *pho7*^+^ stop codon; (iv) an *hygMX* gene (1.69 kbp) conferring resistance to hygromycin; and (v) a 728-bp segment of genomic DNA from nt +2517 to +3245 downstream of the *pho7*^*+*^ start codon. The integration cassettes were excised from the pKS plasmids and transformed into a haploid *S. pombe* strain. Correct insertions were verified by Southern blotting and sequencing of PCR-amplified DNA segments to ascertain the presence of the desired allele. To gauge the effect of these mutations on vegetative growth, cultures of *S. pombe* strains containing the indicated *pho7-TAP* allele were grown in liquid medium until the *A*_600_ reached 0.6 to 0.9. The cultures were adjusted to a final *A*_600_ of 0.1, and aliquots (3 µl) of serial 5-fold dilutions were spotted on YES agar plates, which were then incubated at 18, 20, 25, 30, 34, and 37°C.

### Western blotting.

*S. pombe pho7*Δ and *pho7-TAP* strains were grown in YES medium at 30°C until the *A*_600_ reached 0.6 to 0.8. Aliquots (10 *A*_600_ units) of cells were collected by centrifugation and lysed in 20% trichloroacetic acid. Total acid-insoluble protein was recovered by centrifugation; the pellets were washed with ethanol and resuspended in 1 M Tris-HCl (pH 8.0). Aliquots of the samples, adjusted to contain the same total protein content based on the *A*_280_ of the extracts, were adjusted to 2% SDS and 0.1 M dithiothreitol (DTT) and then analyzed by electrophoresis through 8% polyacrylamide gels containing 0.1% SDS. The gel contents were then transferred to a 0.2-µm-pore polyvinylidene difluoride (PVDF) membrane (Bio-Rad). The membranes were probed by Western blotting with either rabbit polyclonal antibody recognizing the TAP tag (Thermo Fisher) or affinity-purified rabbit polyclonal anti-Spt5 antibody (23). Immune complexes were visualized using horseradish peroxidase-linked anti-rabbit IgG and an ECL (enhanced chemiluminescence) Western detection system (Amersham, GE Healthcare).

### *In silico* search for a candidate Pho7 recognition motif.

Pho7 ChIP-seq data sets were accessed from GEO data sets (accession no. GSE39498) (1). The FASTQ files were mapped to the *S. pombe* genome (ASM294v2.28) using Bowtie-2 2.2.9 (24). The resulting SAM files were converted to BAM files, and all duplicate reads were eliminated using SamTools (25). Pho7 peaks were determined by HOMER (Hypergeometric Optimization of Motif EnRichment) (26). For peak determination, ChIP-seq of Pho7-TAP under phosphate-starved or phosphate-rich conditions was compared to a similarly treated mock immunoprecipitation (IP). Conditions for peak filtering required putative peaks to have normalized sequence read tags 4.5-fold higher than the mock IP. Peak filtering based on local signal required putative peaks to have normalized sequence read tags 3-fold higher than in the surrounding 10-kb region. The sequence within each peak was extracted using a python script (27) and saved as a FASTA file for either Pho7-TAP ChIP in phosphate-replete or phosphate-starved cells. Motif discovery was conducted by MEME (Multiple Em for Motif Elicitation) (8) using the FASTA files. Motifs were predicted within the central 100-nt window of the peak using a zero order background model, with a defined cutoff for minimum-maximum sequence range for a motif to be from 6 to 14 bases with a possibility of any number of motif repetitions. The predicted motif with the lowest E value from both phosphate-replete and phosphate-starved ChIP data was 5′-TCG(C/G)AxTTTxAA. Individual motif occurrences within the sequence of the peaks (FASTA files), conducted by FIMO (Find Individual Motif Occurrences) (9), are listed in Table S1 (cutoff, motif *P* value of ≤0.001). The motifs that correlate with the Pho7 DBD DNase I footprints in the *pho1* and *tgp1* promoters are highlighted in yellow. Annotated genes within 1 kb of the peaks were identified using a python script (27) and are listed in Table S2. The position probability matrices for the motifs predicted by MEME and ChIP peaks identified by HOMER for Pho7 under phosphate-starved or phosphate-rich conditions are given in Table S3 and Table S4, respectively, in the supplemental material.

10.1128/mBio.01218-17.3 TABLE S3  Position probability matrices for the Pho7 motifs predicted by MEME (8) under phosphate-starved (−P_i_) and phosphate-replete (+P_i_) conditions. Download TABLE S3, XLSX file, 0.1 MB. Copyright © 2017 Schwer et al. 2017 Schwer et al. This content is distributed under the terms of the Creative Commons Attribution 4.0 International license.

10.1128/mBio.01218-17.4 TABLE S4  The Pho7 ChIP peaks identified by HOMER (26) are listed on separate pages for phosphate-starved (−P_i_) and phosphate-replete (+P_i_) conditions. Download TABLE S4, XLSX file, 0.1 MB. Copyright © 2017 Schwer et al. 2017 Schwer et al. This content is distributed under the terms of the Creative Commons Attribution 4.0 International license.

### Pho7 DNA-binding domain.

Plasmid pET28b-His_10_-Smt3-Pho7(279–368) encodes the central Zn_2_Cys_6_ domain fused to an N-terminal His_10_-Smt3 module under the transcriptional control of a T7 RNA polymerase promoter. The plasmid was transfected into *E. coli* BL21(DE3) cells. Cultures (2 liters) amplified from single kanamycin-resistant transformants were grown at 37°C in Terrific Broth containing 50 µg/ml kanamycin until the *A*_600_ reached 0.8. The cultures were chilled on ice for 1 h and adjusted to 0.5 mM isopropyl-β-d-thiogalactopyranoside (IPTG) and then incubated for 20 h at 18°C with constant shaking. All subsequent steps of purification were performed at 4°C. Cells were harvested by centrifugation and resuspended in 35 ml buffer A (50 mM Tris-HCl [pH 7.5], 500 mM NaCl, 20 mM imidazole, 10% glycerol). The cells were lysed by sonication, and the insoluble material was removed by centrifugation at 18,000 rpm for 45 min. Supernatants were mixed for 1 h with 4 ml of Ni-nitrilotriacetic acid (NTA) resin (Qiagen) that had been equilibrated with buffer A. The resin was recovered by centrifugation and resuspended in 50 ml buffer A. The washed resin was centrifuged again, resuspended in 50 ml buffer A, and then poured into a column. The bound material was step eluted with buffer A containing 300 mM imidazole. The polypeptide compositions of the flowthrough and eluate fractions were monitored by SDS-PAGE. The 300 mM imidazole eluate fractions containing His_10_-Smt3-Pho7(279–368) were supplemented with Smt3-specific protease Ulp1 and then dialyzed overnight against 2 liters buffer B (50 mM Tris-HCl [pH 7.5], 200 mM NaCl, 10% glycerol). The dialysates were mixed for 1 h with 2 ml of Ni-NTA resin that had been equilibrated with buffer B. Tag-free Pho7(279–368) was recovered in the flowthrough fractions and then subjected to gel filtration through a Superdex-200 column (GE Healthcare) equilibrated in a mixture of 50 mM Tris-HCl (pH 7.5), 200 to 250 mM NaCl, and 10% glycerol. Peak fractions were pooled, concentrated by centrifugal ultrafiltration, and then stored at −80°C at a concentration of 10 mg/ml. Protein concentration was determined as follows. Aliquots of Pho7 DBD and known amounts of bovine serum albumin (BSA) standard were serially diluted and adjusted to 2% SDS and 0.1 M DTT and then resolved by electrophoresis through a 15% polyacrylamide gel containing 0.1% SDS. The gel contents were stained with Coomassie brilliant blue R-250 followed by destaining in a solution containing 22.9% ethanol and 8.4% acetic acid. The gel was scanned, and the staining intensity of the Pho7 DBD and BSA polypeptides was quantified using ImageJ software. A BSA standard curve was generated by plotting intensity as a function of input BSA protein (micrograms); the concentration of Pho7 DBD was calculated by interpolation to the BSA standard curve.

### DNA binding by EMSA.

^32^P-labeled DNA fragments were generated by PCR amplification of *pho1* and *tgp1* promoter segments using 5′ ^32^P-labeled forward primers (prepared with [γ-^32^P]ATP and T4 polynucleotide kinase) and nonlabeled reverse primers. The PCR fragments were purified by electrophoresis through a native 8% polyacrylamide gel in 1× TBE buffer (80 mM Tris-borate, 1.2 mM EDTA), eluted from an excised gel slice, ethanol precipitated, and resuspended in 10 mM Tris-HCl (pH 7.4)–1 mM EDTA at a concentration of 0.6 to 1 µM. Alternatively, 5′ ^32^P-labeled oligonucleotides containing Pho7 binding sites were separated from [γ-^32^P]ATP by Sephadex G25 gel filtration and annealed to a 1.5-fold molar excess of a complementary nonlabeled DNA oligonucleotide in a mixture of 10 mM Tris-HCl (pH 7.4), 1 mM EDTA, and 250 mM NaCl by heating for 20 min at 65°C followed by slow cooling to room temperature. The labeled oligonucleotide duplexes were ethanol precipitated, resuspended in 10 mM Tris-HCl (pH 7.4)–1 mM EDTA–10% glycerol, and then purified by electrophoresis through a native 12% polyacrylamide gel. EMSA reaction mixtures (10 µl) containing 50 mM Tris-HCl (pH 7.4), 10% glycerol, 340 ng poly(dI-dC) (Sigma), 0.6 or 1 pmol ^32^P-labeled DNA (as specified in figure legends), and 2 µl Pho7 DBD (serially diluted in buffer containing 50 mM Tris-HCl [pH 7.5], 250 mM NaCl, 10% glycerol, and 0.1% Triton X-100) were incubated for 10 min at room temperature. The mixtures were analyzed by electrophoresis through a native 6% polyacrylamide gel containing 2.5% (vol/vol) glycerol in 0.25× TBE buffer. ^32^P-labeled DNAs (free and Pho7 bound) were visualized by autoradiography. Where indicated, the extent of DNA binding to Pho7 (as a percentage of total DNA in the sample) was quantified by scanning the gel with a phosphorimager.

### DNase I footprinting.

^32^P-labeled DNA fragments of the *pho1* and *tgp1* promoters were generated by PCR amplification as described above, using either a 5′ ^32^P-labeled forward primer and nonlabeled reverse primer (to label the top strand) or a 5′ ^32^P-labeled reverse primer and nonlabeled forward primer (to label the bottom strand). Footprinting reaction mixtures (10 µl) containing 50 mM Tris-HCl (pH 7.4), 10% glycerol, and ^32^P-labeled DNA and Pho7 DBD as specified were incubated for 10 min at room temperature. The mixtures were then adjusted to 2.5 mM MgCl_2_ and 0.5 mM CaCl and reacted with 0.04 U DNase I (New England Biolabs) for 90 s at room temperature. The DNase I reaction was quenched by adding 200 µl of stop solution (50 mM sodium acetate [pH 5.2], 1 mM EDTA, 0.1% SDS, 30 mg/ml yeast tRNA). The mixture was phenol-chloroform extracted, ethanol precipitated, and resuspended in 90% formamide–50 mM EDTA. The samples were heated at 95°C and then analyzed by urea-PAGE. ^32^P-labeled DNAs were visualized by autoradiography.

### Assay of Pho1 induction during phosphate starvation.

Aliquots of exponentially growing *S. pombe* cultures in YES (yeast extract with supplements) medium were harvested, the cells were washed in water and adjusted to *A*_600_ of ~0.3 in PMG (Pombe glutamate) medium without phosphate. After incubation for 5 h at 30°C, cells were harvested, washed and suspended in water. To quantify Pho1 acid phosphatase activity, reaction mixtures (200 µl) containing 100 mM sodium acetate (pH 4.2), 10 mM *p*-nitrophenylphosphate, and cells (0.02 or 0.1 *A*_600_ unit) were incubated for 5 min at 30°C. The reactions were quenched by adding 1 ml of 1 M sodium carbonate, the cells were removed by centrifugation, and the absorbance of the supernatant at 410 nm was measured. Acid phosphatase activity is expressed in Fig. 1E as the ratio of *A*_410_ (*p*-nitrophenol production) to *A*_600_ (cells). Each data point is the average (±standard error of the mean [SEM]) from three phosphatase assays using cells from three independent cultures.

### Assay of Pho1 plasmid reporter activity.

To measure acid phosphatase activity using the *pho1* (Fig. 5) and *tgp1-pho1* (Fig. 6) plasmid reporters, *pho1*Δ cells, in which the endogenous *pho1* gene is deleted (5), were transfected with the *kanMX*-marked reporter plasmids. Single colonies of G418-resistant transformants (≥20) were pooled, and cultures were grown in YES medium containing G418 (150 µg/ml). Aliquots of exponentially growing cultures were harvested, washed, and suspended in water. Pho1 acid phosphatase activity was quantified as described above. Each data point in the bar graphs in Fig. 5 and 6C is the average (±SEM) from at least three phosphatase assays using cells from at least three independent cultures.
